# Hyperforin Elicits Cytostatic/Cytotoxic Activity in Human Melanoma Cell Lines, Inhibiting Pro-Survival NF-κB, STAT3, AP1 Transcription Factors and the Expression of Functional Proteins Involved in Mitochondrial and Cytosolic Metabolism

**DOI:** 10.3390/ijms24021263

**Published:** 2023-01-09

**Authors:** Alessia Cardile, Valentina Zanrè, Rachele Campagnari, Francesca Asson, Solomon Saforo Addo, Elisa Orlandi, Marta Menegazzi

**Affiliations:** 1 Section of Biochemistry, Department of Neurosciences, Biomedicine and Movement Sciences, University of Verona, Strada Le Grazie, 8, 37134 Verona, Italy; 2 Section of Biology and Genetics, Department of Neurosciences, Biomedicine and Movement Sciences, University of Verona, Strada Le Grazie, 8, 37134 Verona, Italy

**Keywords:** St. John’s wort, cell cycle regulation, apoptosis, ferroptosis, autophagy, STAT3, NF-κB, HIF1α, CREB, metabolism

## Abstract

Hyperforin (HPF), the main component responsible for the antidepressant action of *Hypericum perforatum*, displays additional beneficial properties including anti-inflammatory, antimicrobic, and antitumor activities. Among its antitumor effects, HPF activity on melanoma is poorly documented. Melanoma, especially BRAF-mutated melanoma, is still a high-mortality tumor type and the currently available therapies do not provide solutions. We investigated HPF’s antimelanoma effectiveness in A375, FO1 and SK-Mel-28 human BRAF-mutated cell lines. Cell viability assays documented that all melanoma cells were affected by low HPF concentrations (EC50% 2–4 µM) in a time-dependent manner. A Br-deoxy-uridine incorporation assay attested a significant reduction of cell proliferation accompanied by decreased expression of cyclin D1 and A2, CDK4 and of the Rb protein phosphorylation, as assessed by immunoblots. In addition, the expression of P21/waf1 and the activated form of P53 were increased in A375 and SK-Mel-28 cells. Furthermore, HPF exerts cytotoxic effects. Apoptosis is induced 24 h after HPF administration, documented by an increase of cleaved-PARP1 and a decrease of both Bcl2 and Bcl-xL expression levels. Autophagy is induced, attested by an augmented LC3B expression and augmentation of the activated form of AMPK. Moreover, HPF lowers GPX4 enzyme expression, suggesting ferroptosis induction. HPF has been reported to activate the TRPC6 Ca^++^ channel and/or Ca^++^ and Zn^++^ release from mitochondria stores, increasing cytosolic Ca^++^ and Zn^++^ concentrations. Our data highlighted that HPF affects many cell-signaling pathways, including signaling induced by Ca^++^, such as FRA1, pcJun and pCREB, the expression or activity of which are increased shortly after treatment. However, the blockage of the TRPC6 Ca^++^ channel or the use of Ca^++^ and Zn^++^ chelators do not hinder HPF cytostatic/cytotoxic activity, suggesting that damages induced in melanoma cells may pass through other pathways. Remarkably, 24 h after HPF treatment, the expression of activated forms of the transcription factors NF-κB P65 subunit and STAT3 are significantly lowered. Several cytosolic (PGM2, LDHA and pPKM2) and mitochondrial (UQCRC1, COX4 and ATP5B) enzymes are downregulated by HPF treatment, suggesting a generalized reduction of vital functions in melanoma cells. In line with these results is the recognized ability of HPF to affect mitochondrial membrane potential by acting as a protonophore. Finally, HPF can hinder both melanoma cell migration and colony formation in soft agar. In conclusion, we provide evidence of the pleiotropic antitumor effects induced by HPF in melanoma cells.

## 1. Introduction

Hyperforin (HPF) is an acylphloroglucinol abundantly present in the apical flowers of *Hypericum perforatum,* also known as St. John’s wort (SJW). HPF has been assessed as the main component responsible for the antidepressant action of SJW extract [1].

Considering many experimental results present in the literature, three distinct modes of action can be ascribed to HPF. (i) It can activate the transient receptor potential cation channel (TRPC)-6, resulting in the modulation of synapsis plasticity [2,3,4]. (ii) It can induce calcium and zinc release from mitochondria stores, leading to an increase in their cytosolic concentration [5,6,7]. (iii) Acting as a protonophore, it can change the pH gradient and interfere with the driving force across a membrane (especially of the mitochondria) [8,9]. All these mechanisms may contribute to its biological effects, although each may do so to a different extent, depending on the cell type and the HPF concentrations used [10]. Accordingly, HPF displays a wide spectrum of biological effects besides its antidepressant activity. In the first studies, HPF has been reported to inhibit the activity of cyclooxygenase-1, 5-lypoxygenase and prostaglandin E2-synthase-1 [11,12] and to display antibacterial effects [13]. Subsequently, it was shown that the anti-inflammatory properties of HPF include its capability, in cells, to regulate gene expression and the activation of several cytokine-elicited kinases or transcription factors involved in inflammation [7,14,15,16,17,18] (reviewed by [19,20]). Other studies highlighted SJW and HPF protective effects against noxious stimuli in several animal models as well [21,22,23]. 

Remarkably, Manna et al. demonstrated that a diet with SJW extract can significantly improve the overall survival in a mouse model of azoxymethane-induced colorectal tumorigenesis [24]. In line with this result, a very large epidemiology study indicated that continued use of SJW extract is associated with a 65% decreased risk for colorectal cancer [25], suggesting that it may possess a protective effect in human carcinogenesis as well. 

Importantly, HPF does not possess protective effects only before tumor formation. When tumor mass has already grown, HPF displays remarkable activity against tumor progression by downregulating survival signaling and by inducing programmed cell death. At the beginning of this century, Schempp et al. showed for the first time, in 17 cell lines from different tumor types, that HPF acts by triggering intrinsic apoptotic cell death and inhibiting cell proliferation, with half maximal effective concentration (EC50%) from 3 to 15 µM [26]. Again, the same authors assessed that HPF can hinder tumor growth in rats injected with MT-450 breast carcinoma cells in the absence of any signs of acute toxicity [26]. Subsequently, many studies confirmed the antitumor efficacy of HPF by examining the molecular targets of its cytotoxic effect. 

HPF can affect leukemia cells (reviewed in [27]), such as the human acute promyelocytic leukemia cell line (HL60), by targeting mitochondrial membrane potential [28]. Again, HPF leads to apoptosis in chronic lymphocytic leukemia cells by inducing the pro-apoptotic protein Noxa [29]. HPF affects several acute myeloid leukemia (AML) cell lines, as well as primary cells from AML patients, through different mechanisms including upregulation of Noxa expression, mitochondrial membrane depolarization, activation of caspases and inhibition of protein kinase B (AKT) activity [30].

Regarding solid tumors, Hsu et al. showed that HPF can induce apoptosis in glioblastoma cells, with EC50% of 5–10 µM, by suppressing the expression of antiapoptotic-related proteins. Furthermore, they demonstrated that HPF can inactivate epithelial growth factor receptor (EGFR), extracellular signal-regulated kinases (ERK)1/2, and nuclear factor kappa B (NF-κB) [31]. HPF can inhibit cyclin D1 expression and can induce the loss of internal mitochondrial membrane potential in hepatocarcinoma cells [32]; in addition, it can affect tumor growth of non-small-cell lung cancer [33]. HPF decreases bladder cancer cell survival, with EC50% of 10–20 µM, by increasing reactive oxygen species (ROS), calcium signaling and apoptosis and by blocking NF-κB activity [34]. Recently, cytotoxicity of HPF and its derivatives was discovered in colon cancer cells by a mechanism involving the Wnt/β-catenin signaling pathway [35]. Donà et al. studied the HPF-elicited cytotoxicity in many human and murine cell lines [36]. They found that HPF decreases ERK1/2 and metalloproteinases activity and affects cell viability at a concentration in the range of 5–20 µM, whereas untransformed endothelial cells were only marginally affected. Importantly, the authors reported that HPF concentrations 20-fold below the toxicity threshold were effective in hindering the invasive potential of malignant cells. Moreover, in mice injected with neoplastic cells, HPF administration reduced tumor growth and metastasis with persistence of healthy behavior [36]. 

Regarding melanoma, the HPF molecular mechanism has not yet been investigated, although its effectiveness in reducing the cell viability of some human or mouse melanoma cell lines has been previously reported [26,36,37]. 

Malignant melanoma is an aggressive skin tumor characterized by high metastatic potential and mortality [38]. In about 40–60% of patients with cutaneous melanoma, oncogenic mutations in BRAF kinase are found, which drive the constitutive activation of pro-survival kinases ERK1/2 [38]. Remarkably, patients harboring BRAF mutations showed poorer prognosis and survival [39]. BRAF inhibitors and mitogen-activated protein kinase (MEK)-inhibitors therapies are able to improve the lifespan of patients accompanied with the BRAF-mutated genotype [40]. Unfortunately, the effectiveness of targeted therapy has been shown to be time-restricted because melanoma becomes resistant to these drugs in a short time [41]. Therefore, new strategies to extend patients’ survival should be discovered.

In the present work, three BRAF-mutated human melanoma cell lines are tested for their sensitivity to HPF administration. Data show that all cell lines are significantly affected by low µM concentration of HPF in contrast to normal human epithelial melanocytes. In addition, we documented pleiotropic effects by which HPF counteracts melanoma cell malignancy. 

## 2. Results

### 2.1. A375, FO-1, SK-Mel-28 and MeWo Melanoma Cell Viability Was Affected by Hyperforin in a Time- and Concentration-Dependent Manner

**Figure 1:**
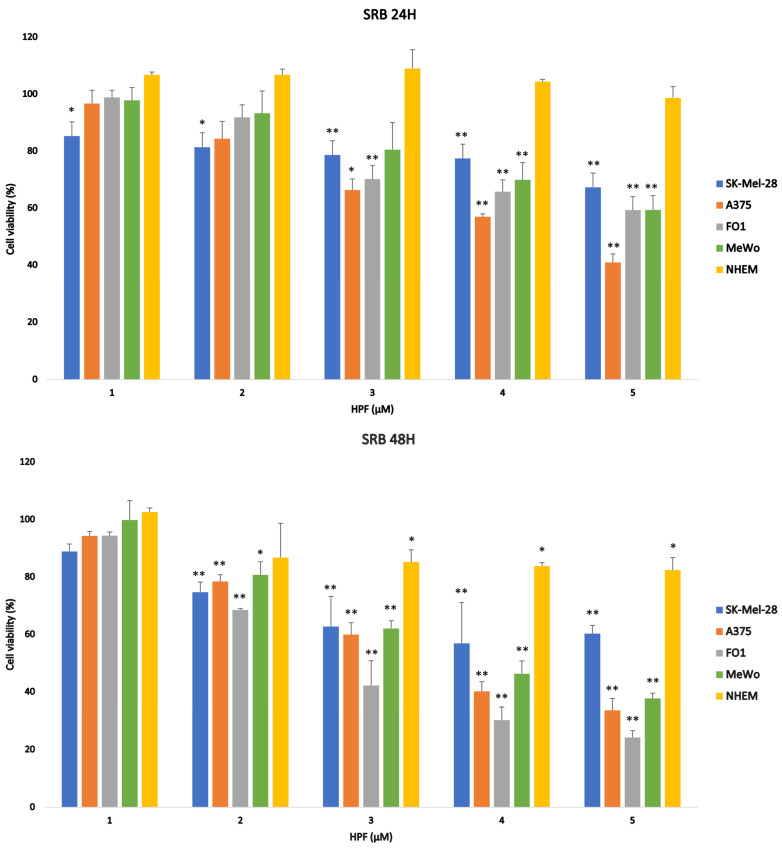

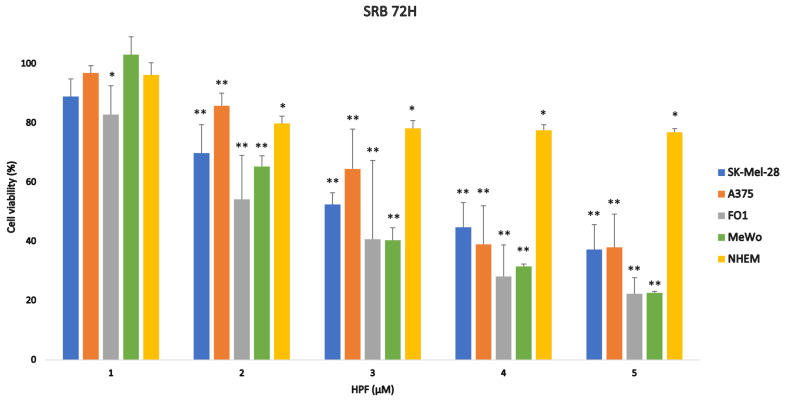
Histograms show time- and concentration-dependent reduction in cell viability after hyperforin treatment. SRB cell viability assay was performed on A375 (orange), FO-1 (grey), SK-Mel-28 (blue) and MeWo (green) melanoma cells treated with 1 to 5 µM hyperforin (HPF) for 24, 48 and 72 h. HPF induced a time- and concentration-dependent decrease in cell viability, in comparison with untreated cells (100%). Normal human epithelial melanocytes (NHEM, yellow), treated with HPF for 24, 48 and 72 h, was shown to be less affected by HPF than melanoma cells. Data acquired calculating the average ± S.D. of values obtained from at least four independent experiments were compared with the untreated control (* *p* < 0.05; ** *p* < 0.01).

To quantify the antitumor effect of HPF on melanoma cells, a sulforhodamine B (SRB) viability assay was carried out on SK-Mel-28, A375 and FO-1 BRAF-mutated melanoma cells and on P53-mutated MeWo cells after 24, 48 and 72 h of treatment with 1, 2, 3, 4 and 5 µM HPF. Results shown in Figure 1 reveal an evident time-dependent inhibition of cellular mass. Indeed, the effective HPF concentration able to achieve 50% cell viability (EC50%) of untreated cells was in a range from 2 to 4 µM after 48 or 72 h treatments (Figure 1). FO-1 was the most responsive melanoma cell line to HPF administration, with an EC50% of 2 µM after 72 h, while A375, SK-Mel-28 and MeWo cells displayed an EC50% of about 3–4 µM (Figure 1).

To investigate whether normal human epithelial melanocytes (NHEM) were affected by HPF treatment, increasing concentrations of HPF were added to NHEM culture medium and a cell viability assay was carried out after 24, 48 and 72 h. Results indicate that HPF at 5 µM, the highest concentration used on melanoma cells, reduces NHEM viability by 20% in comparison to a 70–80% reduction obtained in malignant cell lines (Figure 1).

Therefore, data suggest that HPF affects melanoma cell viability with higher specificity than normal melanocytes. 

### 2.2. Hyperforin Affects the Morphology of Melanoma Cells

Morphological features acquired by melanoma cells after 24 and 48 h treatment with low micromolar concentrations of HPF were analyzed by microscopy examination. 

**Figure 2:**
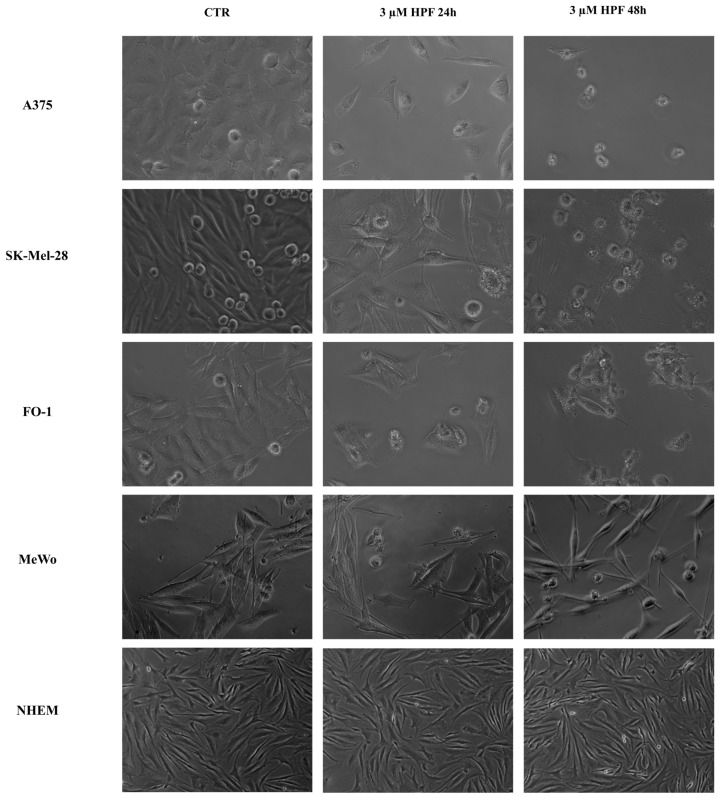
The images show melanoma cell morphological modifications after 24 and 48 h-treatment with 3 µM hyperforin. Representative images of A375, SK-Mel-28, FO-1 and MeWo melanoma cells and normal human epithelial melanocytes (NHEM), untreated (CTR) or treated with 3 µM hyperforin (HPF) for 24 and 48 h. In melanoma cells but not in NHEM, HPF induces a time-dependent decrease in cell number and a change in cell shape from elongated to round with membrane blebbing. Images were captured at 20× magnification (5× magnification only for NHEM) with an inverted microscope (Axio Vert A1, Zeiss, Oberkochen, Germany).

Figure 2 shows that HPF can affect the natural shape of A375, FO-1, SK-Mel-28 and MeWo melanoma cells in 2D culture. Control cells turn out to be well adherent to the plate except for several cells in division showing rounded and translucent shapes (Figure 2, CTR). After 24 h treatment with 3 µM HPF, dividing cells as well as the total cell number were decreased. At 24/48 h after HPF administration, almost all remaining cells had lost adherence to the plate and several cells showed blebbing of the plasma membrane, a typical morphology of cells undergoing late phase of apoptotic cell death (Figure 2). No change in morphology was detectable in NHEM after 24–48 h of HPF treatment (Figure 2).

### 2.3. Hyperforin Inhibits Melanoma Cell Proliferation by Affecting the Expression of Cell Cycle-Regulating Proteins

SRB results ascertained that HPF can reduce total cell mass in melanoma cells, and morphological analysis suggested its effect on both cell proliferation and death. In order to investigate the HPF mechanism of action, we selected the most homogeneous cell lines presenting the mutation in the BRAF gene to perform all the following experiments.

Firstly, a cell proliferation assay was carried out by measuring Br-deoxy-uridine (BrdU) incorporation during the cell cycle DNA synthesis (S) phase. A375, FO-1 and SK-Mel-28 melanoma cells were treated with 2, 3 and 4 µM HPF for 48 h. At that time, BrdU was added to the culture medium and, after 8 h incubation, its incorporation in newly synthesized DNA was measured.

**Figure 3:**
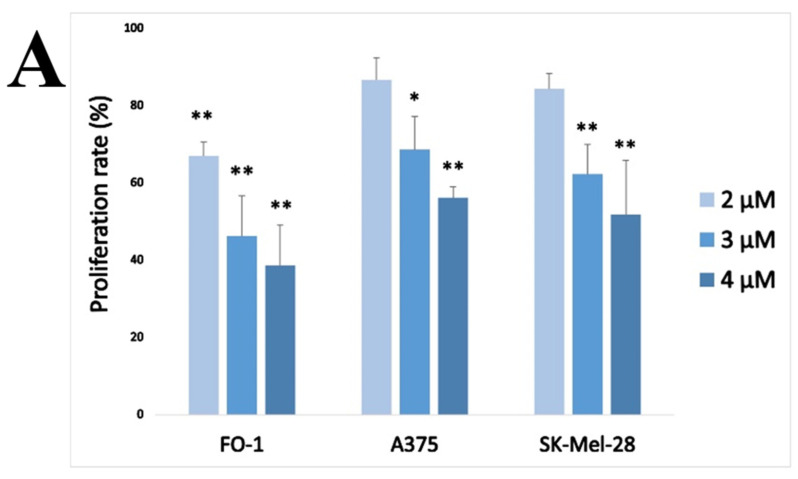

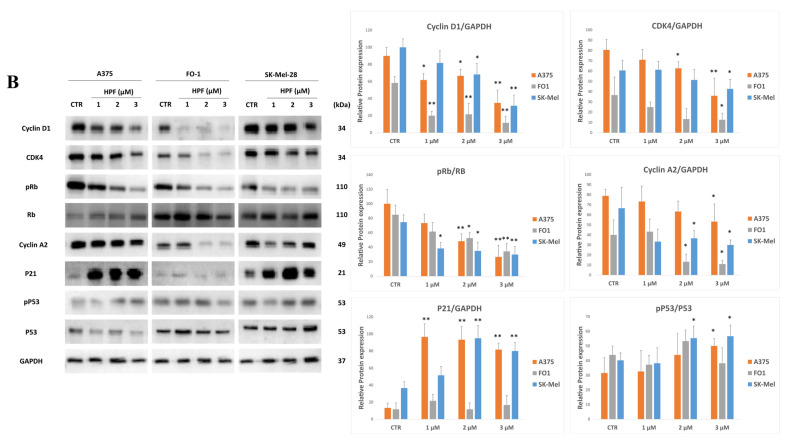
Hyperforin slows down melanoma cell proliferation by affecting the expression of several cell cycle-regulating proteins. (**A**) BrdU incorporation assay attesting the inhibition of cell proliferation of 2, 3 and 4 µM HPF on FO-1, A375 and SK-Mel-28 melanoma cell lines. (**B**) A375, FO-1 and SK-Mel-28 melanoma cells were treated with increasing concentrations of HPF for 24 h. In the left panel, representative immunoblots show the expression level of cyclin D1, cyclin-dependent kinase (CDK)-4, the phosphorylated form of retinoblastoma protein (pRb), total Rb protein, cyclin A2, P21/WAF1 (P21), phospho-Ser15-P53 (pP53), total P53 (P53) and glyceraldeide-3-phosphate dehydrogenase (GAPDH). On the right, histograms represent the mean values ± S.D. of protein expression level measured by densitometry deriving from three independent experiments and normalized with GAPDH expression, whereas pRb and pP53 results were normalized with total Rb and P53 protein level, respectively. All comparisons were performed vs. each control sample after data normalization; * *p* < 0.05; ** *p* < 0.01.

As shown in Figure 3A, HPF reduced A375, FO-1 and SK-Mel-28 cell proliferation rates. This result led us to investigate the underlying molecular mechanisms by immunoblots, carried out to discover the expression levels of target proteins controlling the cell cycle.

Cyclin D1 together with cyclin-dependent kinase (CDK)-4 are key regulators of the G1 phase of the cell cycle, and they are considered therapeutic targets in BRAF-mutated cancers [42]. Immunoblots showed a concentration-dependent decrease of cyclin D1 and CDK4 expression level in melanoma cells treated for 24 h with HPF (Figure 3B). Retinoblastoma (Rb) protein represents a major G1 restriction point aimed to block cell entry in S-phase. Cyclin D1/CDK4 activation determines hyperphosphorylation and inhibition of Rb protein, allowing cell cycle progression [43]. The phosphorylation level of Rb (pRb) was decreased in a concentration-dependent manner by a 24 h HPF treatment, whereas the total Rb protein level was unchanged (Figure 3B). Cyclin A2 is highly expressed in the S-phase when it is associated with CDK2 or CDK1 [44]. In addition to cyclin D1, cyclin A2 expression level was decreased after 24 h of treatment (Figure 3B). The cell cycle progression inhibitor P21/Waf1 (P21) protein can bind cyclin A2/CDK2 and cyclin D1/CDK4 complexes by hindering their activity. P21 protein binding makes cyclin D1/CDK4 complex inactive and unable to phosphorylate Rb protein [45]. Remarkably, P21 expression level was maximally increased after 24 h treatment in A375 and SK-Mel-28 cell lines (Figure 3B). Tumor suppressor P53 is a transcription factor able to induce the expression of many genes, including P21 protein, inhibiting, finally, cell cycle progression [46]. Immunoblots showed an increased expression of the active form of P53 (phospho-Ser15-P53) in the P53 wild type A375 and SK-Mel-28 cell lines concomitantly with an increase of P21 expression (Figure 3B). Instead, FO-1 cells expressed a very low level of P21 and the amount of P53 phosphorylation remains unchanged until 2 µM HPF, decreasing at higher concentrations (Figure 3B).

In summary, all data suggest a pleiotropic mechanism elicited by HPF able to hinder cell cycle progression in melanoma cells.

### 2.4. Hyperforin Induces Apoptotic Cell Death, as Well as Autophagy and Ferroptosis

**Figure 4:**
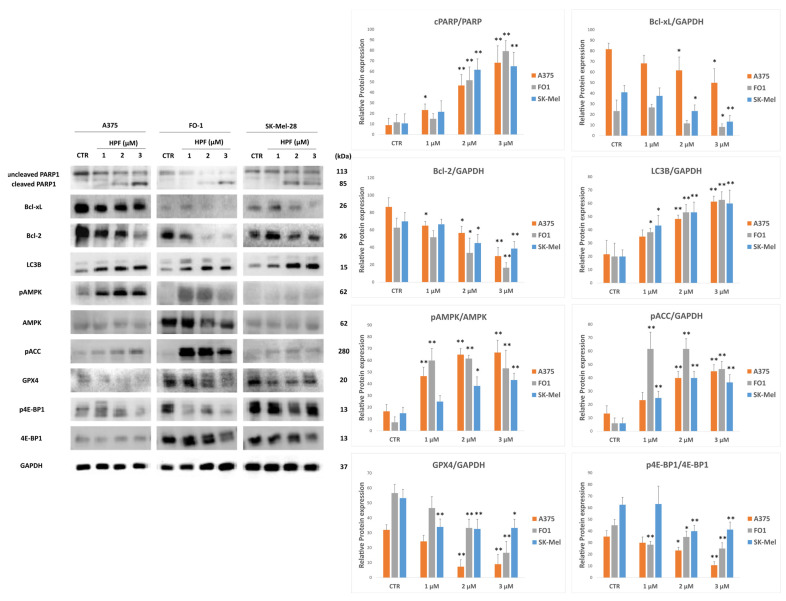
Hyperforin affects the expression of several proteins involved in apoptosis, autophagy and ferroptosis induction. A375, FO-1 and SK-Mel-28 melanoma cells were treated with increasing concentrations of HPF for 24 h. In the left panel, representative immunoblots show the expression level of cleaved and uncleaved PARP1, Bcl-extra-large (Bcl-xL), Bcl2, microtubule-associated proteins 1A/1B light chain 3B (LC3B), the phosphorylated form of AMP-activated kinase (pAMPK), total AMPK, the phosphorylated form of acetylCoA carboxylase (pACC), glutathione peroxidase 4 (GPX4), the phosphorylation level of eIF4E-binding protein 1 (4E-BP1), total 4E-BP1 and glyceraldeide-3-phosphate dehydrogenase (GAPDH). On the right, histograms represent the mean values ± S.D. of protein expression level measured by densitometry deriving from three or more independent experiments and normalized with GAPDH expression, unless otherwise stated. All comparisons were performed vs. each control sample after data normalization; * *p* < 0.05; ** *p* < 0.01.

Morphological analysis of melanoma cells showed several signs of cell death at 24 and 48 h after HPF administration (Figure 2). Therefore, we analyzed some molecular markers of programmed cell death. The cleaved form of poly (ADP-ribose) polymerase 1 (cleaved-PARP1) is obtained through the activity of caspase 3 or caspase 7 in cells undergoing apoptosis [47,48]. After 24 h of HPF treatment, in all melanoma cell lines, immunoblots showed a decrease of bands representing full-length PARP1 expression and a correspondent increase of higher mobility band displaying cleaved-PARP1 level (Figure 4). Thereby, data suggest induction of apoptotic cell death in a concentration-dependent manner. Proteins belonging to the B-cell lymphoma 2 (Bcl2) family are regulators of apoptosis. Bcl2 and Bcl-extra-large (Bcl-xL) are antiapoptotic proteins highly expressed in tumor cells [49,50]. Immunoblots showed a gradual decrease of both Bcl2 and Bcl-xL expression levels (Figure 4), explaining in part the high propensity of cells treated for 24 h with HPF to undergo apoptosis. 

Then, the involvement of other types of programmed cell death was investigated. It has been reported that HPF augments AMP-activated kinase (AMPK) activity either in normal [51] or in cancer cells [28]. Immunoblot results showed an increase of the phosphorylated and activated form of AMPK (pAMPK) after 24 h of HPF treatment, whereas the total level of the kinase was not significantly affected (Figure 4). In agreement with the increased pAMPK level, acetylCoA carboxylase enzyme (ACC), which is a known target of AMPK activity, has been found phosphorylated as well (pACC, Figure 4). AMPK is an activator of mitophagy/autophagy in tumor cells, including melanoma [52]. Therefore, the expression level of microtubule-associated proteins 1A/1B light chain 3B (LC3B), a known marker of autophagy, was investigated by immunoblotting. Data show that LC3B increased after HPF treatment (Figure 4), suggesting activation of autophagy as well.

Glutathione peroxidase (GPX)-4 is an essential regulator of ferroptotic cell death since its knockdown or its overexpression can induce or antagonize, respectively, this form of cell death [53,54]. After 24 h treatment with HPF, the expression level of GPX4 enzyme was significantly reduced (Figure 4). Altogether, these data could suggest a co-presence of ferroptosis. 

Finally, the phosphorylation level of eIF4E-binding protein 1 (4E-BP1) displayed a decrease, demonstrating that 24 h HPF treatment induces generalized translational repression in cells (Figure 4). 

### 2.5. Signaling Pathway Elicited by Hyperforin in Melanoma Cells

After establishing its antitumor activity, the mechanisms involved in the HPF-elicited cytotoxicity in melanoma cells were investigated.

As already mentioned, it has been reported that HPF acts as a specific activator of Ca^++^-channel TRPC6 [2], it induces an increase of cytosolic Ca^++^ and Zn^++^ concentrations [5] and it facilitates proton flux between cell membranes acting as a protonophore [9].

Experiments were performed to verify if the Ca^++^ channel blocker SKF-96365 (SKF) could suppress the cytostatic/cytotoxic effects of HPF in melanoma cells. In addition, BAPTA and TPEN, the cell-permeable chelators of Ca^++^ and Zn^++^, respectively, were used to ascertain whether the blockage of the HPF-elicited Ca^++^ and Zn^++^ cytosolic concentrations’ increase could suppress its antimelanoma activity. Thus, SRB viability assay was carried out on melanoma cells treated for 72 h with SKF, BAPTA or TPEN alone and in the co-presence of HPF, at concentrations corresponding approximately to the EC50% value measured in each cell line. All inhibitors were added to the cell culture medium 10 min before HPF addition.

The obtained data were very consistent considering the different sensitivity of each melanoma cell line to HPF. 

**Figure 5:**
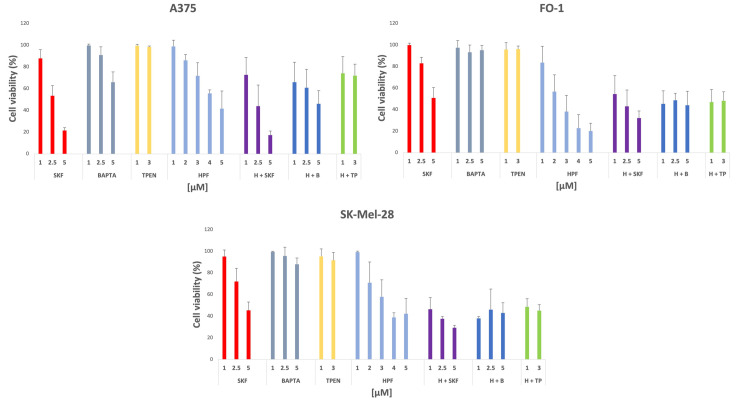
Histograms show the reduction in cell viability in presence of SKF, BAPTA and TPEN administered alone or in co-presence of hyperforin. SRB cell viability assay was performed on FO-1, SK-Mel-28 and A375 melanoma cells after 72 h of treatment with SKF-96365, an inhibitor of Ca^++^-channel transient receptor canonical (TRPC)-6, or BAPTA-AM, a cell-permeable Ca^++^ chelator, or TPEN, a Zn^++^ chelator. The co-treatment was performed with each inhibitor singly, combined with HPF used at 2 µM for FO-1 and at 4 µM for A375 and SK-Mel-28 cells, accordantly with each EC50% value previously documented. Data acquired calculating the average ± S.D. of values were obtained from at least four independent experiments.

As shown in Figure 5, the treatment with SKF alone at the lowest concentration used (1 µM) did not affect cell viability. Furthermore, SKF administration in association with HPF was not able to revert the cytostatic/cytotoxic activity of this acylphloroglucinol. Instead, the use of higher SKF concentrations alone (2.5 and 5 µM) induced a noticeable reduction of cell viability after 72 h of treatment. Remarkably, at these doses, HPF in association with SKF can increase, rather than hinder, its harmful effect in all melanoma cell lines (Figure 5).

Again, cell viability was slightly affected by 1, 2.5 or 5 µM of BAPTA or by 1 or 3 µM of TPEN administered singly to the culture medium. Moreover, the co-presence of HPF with BAPTA or TPEN did not block the HPF cytostatic/cytotoxic effect (Figure 5). 

Thus, for a longer time of treatment (72 h), data show that the antimelanoma activity of HPF was maintained even in the presence of either a TRPC6 blocker or a Ca^++^ or Zn^++^ chelator.

**Figure 6:**
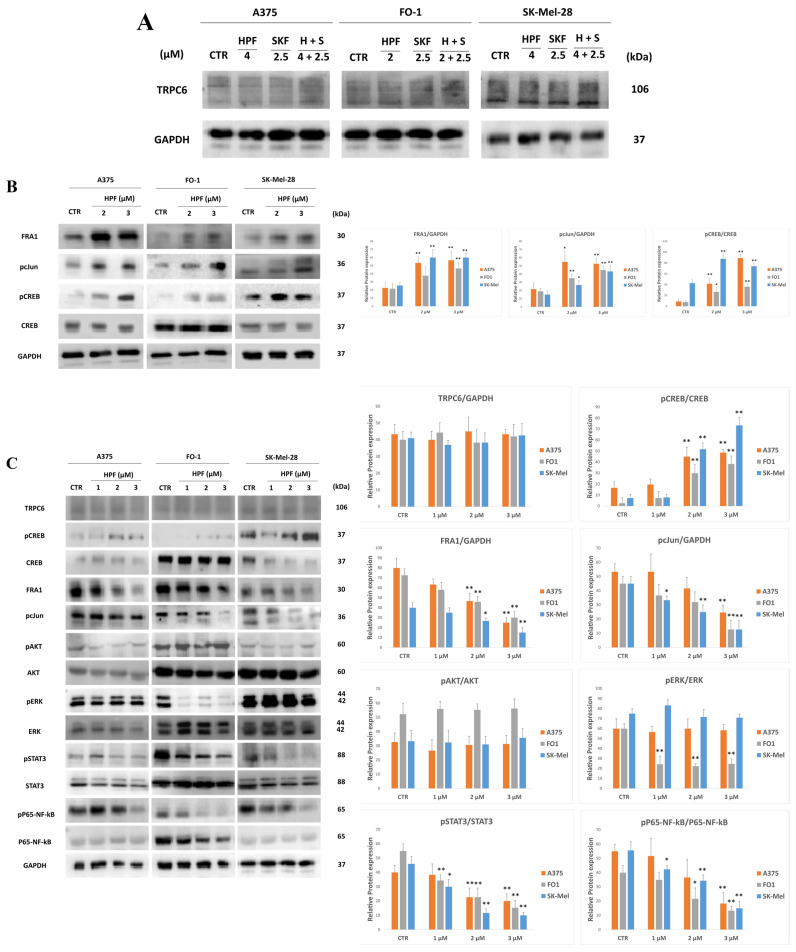
Hyperforin affects the expression levels of key proteins involved in signaling. (**A**) Represents immunoblots showing the expression level of TRPC6 in A375, FO-1 and SK-Mel-28 melanoma cells treated for 2 h with HPF, SKF, or both compounds. (**B**) Represents immunoblots showing the expression level of Fos-related antigen 1 (FRA1), the phosphorylated form of proto-oncogene c-Jun (pcJun) and of cyclic AMP response-element binding protein (pCREB), and total CREB in melanoma cells treated for 2 h with 0, 2 and 3 µM HPF concentrations. On the right, histograms represent the mean values ± S.D. of protein expression levels measured by densitometry deriving from three independent experiments and normalized with GAPDH expression, unless otherwise stated. All comparisons were performed vs. each control sample after data normalization; * *p* < 0.05; ** *p* < 0.01. (**C**) Represents immunoblots showing the expression level of key proteins belonging to several signaling pathways activated in melanoma cells after 24 h HPF treatment. In particular, the expression of TRPC6, pCREB, total CREB, FRA1, pcJun, the phosphorylated form of protein kinase B (pAKT), total Akt, the phosphorylated form of extracellular signal-regulated kinases (ERK), total ERK, the phosphorylated form of signal transducer and activator of transcription 3 (STAT3), total STAT3, the phosphorylated form of nuclear factor kappa B P65 subunit (pP65 NF-κB), total P65 NF-κB and GAPDH. On the right, histograms represent the mean values ± S.D. of protein expression levels measured by densitometry deriving from three independent experiments and normalized. All comparisons were performed vs. each control sample after data normalization; * *p* < 0.05; ** *p* < 0.01.

In addition to its effect on TRPC6 channel activity, HPF could affect the expression level of the TRPC6 channel. Immunoblotting data showed that TRPC6 protein level was unchanged after 24 h treatment with HPF (Figure 6). In cells treated with HPF, SKF or both compounds for a short time (2 h), TRPC6 expression was unchanged as well (Figure 6). Data suggest that TRPC6 was not overexpressed after HPF treatment.

At the molecular level, in different cancer models, several reports attested that many signaling pathways are affected by HPF administration (reviewed in [55]). Gibon et al. [7] reported that, in some tissues, HPF promotes the activation of the transcription factor cyclic-AMP response element-binding protein (CREB). In agreement with these previous data, in all the analyzed melanoma cell lines, 2 and 24 h after HPF administration, immunoblots showed a concentration-dependent increase of the phosphorylated and activated form of CREB (pCREB), whereas total CREB expression was unchanged or even decreased (Figure 6B,C). 

After short time treatments, HPF was also able to increase the expression of proteins belonging to the transcription factor complex activating protein-1 (AP1). Immunoblots showed that HPF concentration dependently increases Fos-related antigen 1 (FRA1) expression and the phosphorylated and activated form of the proto-oncogene cJun (pcJun) (Figure 6B). However, after 24 h treatment, FRA1 and pcJun expression were decreased in a concentration-dependent manner in all the tested cell lines (Figure 6C). 

Merhi et al. showed [30] a suppression of protein kinase B (AKT) activity in human myeloid cells at HPF concentrations similar to those used in the present work, whereas Hsu et al. proved that HPF can inhibit the extracellular signal-regulated kinases (ERK)-1/2 activity in glioblastoma cells [56]. Regarding pERK1/2 and pAKT, which are both constitutively active in the BRAF-mutated melanoma cell lines, no different expression levels were found at short times (not shown) and after 24 h treatment in A375 and SK-Mel-28 cells (Figure 6C). In FO-1 cells only, a reduction of pERK1/2 level was found 24 h after HPF administration (Figure 6C).

Key transcription factors largely involved in pro-survival signaling in melanoma are signal transducer and activator of transcription (STAT)-3 [57] and nuclear factor-kappa B (NF-κB) [58], which were shown to be activated by phosphorylation in melanoma cells. After 24 h of treatment, HPF concentration dependently decreased the expression levels of phospho-Ser475 of P65-NF-κB subunit and phospho-Tyr705 of STAT3, while total P65 and total STAT3 protein levels were unchanged (Figure 6C).

### 2.6. Metabolic Effects of Hyperforin in Melanoma Cells

**Figure 7:**
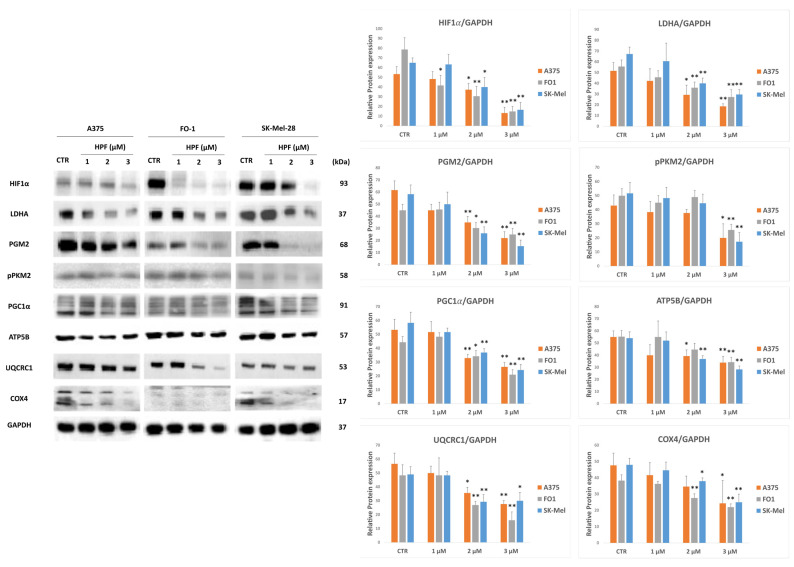
Hyperforin affects the expression levels of key proteins involved in cytosolic and mitochondria metabolic pathways. Represented immunoblots show the expression level of key proteins involved in melanoma cells metabolism after 24 h HPF treatment. In particular, the expression of hypoxia-inducible factor (HIF)-1α, lactate dehydrogenase A (LDHA), phospho-gluco-mutase2 (PGM2), the phosphorylated form of pyruvate kinase M2 (pPKM2), peroxisome proliferator-activated receptor-gamma coactivator (PGC)-1α, c1 subunit of the ubiquinol cytochrome c reductase (UQCRC1), ATP synthase F1 subunit β (ATP5B), cytochrome c oxidase subunit IV (COX4) and GAPDH. On the right, histograms represent the mean values ± S.D. of protein expression levels measured by densitometry deriving from three independent experiments and normalized. All comparisons were performed vs. each control sample after data normalization; * *p* < 0.05; ** *p* < 0.01.

Cancer cells are able to reprogram their metabolism by switching from the mitochondrial oxidative pathway (OXPHOS) to glycolytic anaerobic metabolism and vice versa [59]. The transcription factor hypoxia-inducible factor (HIF)-1α, among others, can control several enzymes regulating metabolic flux [59]. Firstly, we measured the HIF1α expression level after 24 h of HPF treatment. HIF1α protein level was decreased in all melanoma cells (Figure 7). Consistently, some cytosolic enzymes, such as lactate dehydrogenase A (LDHA), phosphoglucomutase 2 (PGM2) and the phosphorylated form of pyruvate kinase M2 (pPKM2) were downregulated (Figure 7).

Thus, expression levels of mitochondrial key proteins were measured. The expression level of peroxisome proliferator-activated receptor-gamma coactivator (PGC)-1α, which is a transcription factor stimulating mitochondria biogenesis, was decreased after 24 h treatment with HPF, as well as the expression of c1 subunit of the ubiquinol cytochrome c reductase (UQCRC1) and ATP synthase F1 subunit β (ATP5B). The protein level of the cytochrome c oxidase subunit IV (COX4) was increased 30 min after HPF addition (not shown), but its expression decreased after 24 h (Figure 7).

In summary, the expression levels of proteins regulating both cytosolic and mitochondrial metabolism are lowered by HPF treatment in all melanoma cells.

### 2.7. Hyperforin Affects the Rate of Cell Migration of FO-1 and SK-Mel-28 Melanoma Cells and It Hinders the Ability of Melanoma Cells to Form Colonies in Soft Agar

The wound healing assay is widely used to measure cell migration. The method is based on quantifying the rate at which cells can close an artificial scratch created in a confluent cell monolayer. 

**Figure 8:**
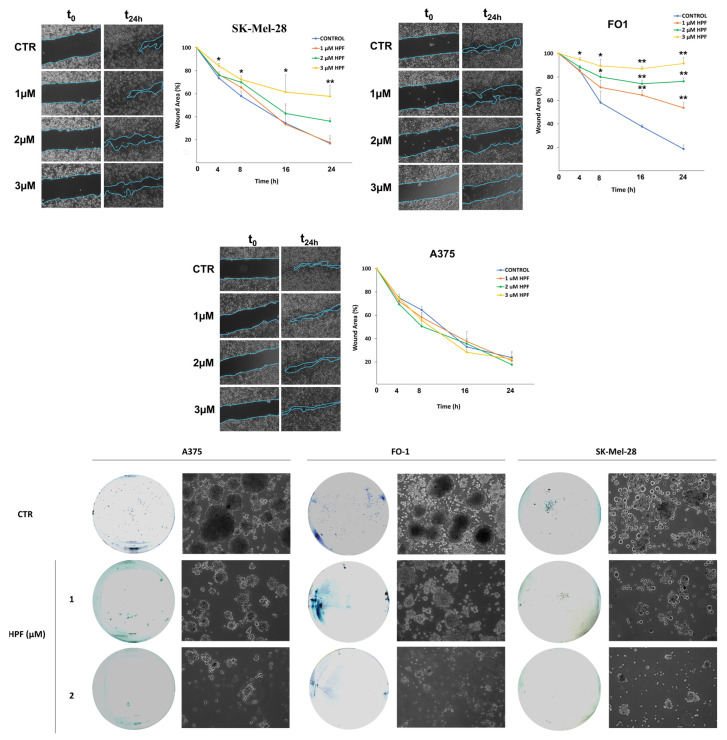
Hyperforin concentration dependently affects cell mobility and inhibits soft agar colony formation in melanoma cell lines. **Top**, wound healing assay attesting the inhibition of cell mobility by 2 and 3 µM HPF in FO-1 and SK-Mel-28 melanoma cells but not in A375 cell line. Images were recorded with an inverted microscope (Axio Vert A1, Zeiss, Oberkochen, Germany). The images were analyzed quantitatively using ImageJ computing software (NIH Image, Bethesda, MD, USA). **Bottom**, images of colonies taken from representative experiments of colonies formed in soft agar. Representative images showing growth and density of colonies were taken by an inverted microscope (5× magnification). * *p* < 0.05; ** *p* < 0.01.

Figure 8 shows the ability of 2 and 3 µM HPF to slow down the migration rate of FO-1 and SK-Mel-28 melanoma cells. Instead, HPF did not delay the wound healing closure of the A375 cell line, although it decreased the density of cells filling the wound in comparison with the untreated sample (Figure 8).

To investigate if HPF can inhibit melanoma cell growth in an anchorage-independent manner, a soft agar colony formation assay was performed. Both 1 and 2 µM HPF can decrease the number and dimension of colonies formed in soft agar after 14–21 days of culture (Figure 8).

## 3. Discussion

HPF is the principal and active component of SJW extract, commonly used as an antidepressant medicament by many people around the world. In addition to this proven effect, SJW extract and HPF display additional beneficial properties including anti-inflammatory, antimicrobic and antitumor activities (reviewed by [19,20,55]). The large HPF bioavailability, the persistence of its protective benefits and the absence of adverse effects makes this compound suitable for both tumor prevention [25,60] and treatment [55]. 

Among the studies that investigate the antitumor activity of HPF, its mechanism of action on melanoma cells is poorly documented. Melanoma is still a high-mortality tumor type and the therapies currently available are not conclusive [38]. Therefore, we investigated HPF effects on three melanoma cell lines harboring the dangerous but frequent mutation in the BRAF gene, which makes downstream proliferative signaling pathways constitutively active [38,40]. The HPF antimelanoma activity was also documented on a BRAF wild-type but P53-mutated MeWo melanoma cell line. 

Independently of their mutations, all melanoma cell lines tested were very responsive to the damaging effects of HPF, as their viability was affected by EC50% HPF concentrations in the range of 2–4 µM (Figure 1), lower than those previously registered in many other non-melanoma tumor cells [26,34,35,36,56]. Subsequently, we investigated the mechanism of HPF antimelanoma activity on the three BRAF-mutated cell lines. 

In A375, FO-1 and SK-Mel-28 cells, HPF displays cytostatic activity hindering cell cycle progression, as demonstrated by the lower BrdU incorporation in comparison with untreated control cells (Figure 3A). HPF seems to move towards several targets in the cell cycle because it turns out to be able to decrease cyclins D1 and A2 and CDK4 protein expression (Figure 3B). These results are in line with the findings of Liu et al. [34], who registered a suppression of cyclin D1 expression in a human bladder cancer cell line. It is very significant that the activation of the cyclin D1/CDK4 complex passes through the G1/S checkpoint by triggering hyperphosphorylation of the Rb protein [43]. Notably, pRb expression level is concentration-dependently decreased by HPF, despite a stable total Rb protein level (Figure 3B), suggesting an early blockage of the cell cycle. In addition, HPF inhibits the expression of cyclin A2 that acts downstream of the G1/S checkpoint. At the same time HPF, driving a maximal induction of P21/Waf1 expression, would provide for a generalized blockage of all the phases of the cell cycle, since P21 can inhibit, in addition to the cyclin D1/CDK4 complex, the activity of cyclin E/CDK2, cyclin A2/CDK2 and cyclin B/CDK1 complexes [45]. P21 expression can be elicited in a P53-dependent or independent manner [46]. We found, indeed, a concomitant increase of P53 phosphorylation level in A375 and SK-Mel-28 but not in FO-1 melanoma cells. The activation of AMPK by phosphorylation, as we showed in Figure 4, can also participate in P21 induction, as previously reported by Ma et al. and Petti et al. [61,62].

In addition to cell cycle arrest, HPF induces a cytotoxic effect in melanoma cell lines, visible through cellular morphology analysis which highlighted several signs of cell death at both 24 and 48 h after treatment (Figure 2). Immunoblots showed a concentration-dependent increase of the cleaved PARP1 band and a concomitant decrease of its full-length band, in all BRAF-mutated melanoma cell lines (Figure 4). This evident marker of apoptotic cell death was accompanied by a decrease in expression levels of the antiapoptotic proteins Bcl2 and Bcl-xL (Figure 4). Many other authors reported that apoptosis is induced by HPF in different tumor types, with mechanisms involving other pro- or antiapoptotic effector proteins [29,30,32,56], or by mediating the Bcl2/Bcl-xL axis [63,64]. 

We also showed autophagic cell death, the marker of which, LC3B, was affected by HPF treatment (Figure 4). This result is in line with both the increase of AMPK phosphorylation (Figure 4), an autophagy activator, and with data reported by Wiechmann et al., obtained in HL60 leukemic cells [28]. In a non-tumor context, HPF was recently reported to trigger thermogenesis in adipose tissue by activating autophagy via AMPK [51]. Notably, activated AMPK can inhibit the mechanistic target of the rapamycin (mTOR) complex, blocking the phosphorylation of its downstream substrate 4E-BP1, leading to protein synthesis repression [65]. Indeed, we found a hypo-phosphorylated 4E-BP1, which is unable to promote protein synthesis (Figure 4).

One novelty is the possible involvement of another form of regulated cell death, called ferroptosis, characterized by lipid peroxidation induced mainly by free iron. The principal molecular maker of ferroptosis is the antioxidant enzyme GPX4, whose depletion allows death process. Indeed, GPX4 knockdown triggers cell death accompanied by lipid peroxidation [53,54]. Treatment with HPF for 24 h decreased GPX4 expression level in a concentration-dependent manner (Figure 4). Although the involvement of ferroptosis needs further investigation, we could hypothesize that, after HPF-elicited depolarization of the mitochondrial inner membrane triggered by its activity as a protonophore, mitophagy/autophagy can be induced to remove injured mitochondria. Mitophagy/autophagy is reported to increase free iron through ferritin degradation, subsequently eliciting ferroptosis [66]. Indeed, the knockdown of ATG5, which is indispensable for autophagy, inhibits ferroptosis as well [54,66]. In this manner, autophagy and ferroptosis can both be induced in the same cell. Data suggest that HPF induces not only apoptosis but also other regulated cell deaths, such as autophagy and ferroptosis, with a mechanism that will be deeply investigated in the future.

For its extreme lipophilicity (XlogP 9.6), HPF does not need any membrane receptor to enter cells. Nevertheless, we considered the possibility that HPF’s antitumor effect could depend on the binding and subsequent activation of the TRPC6 Ca^++^ channel, as previously reported by other authors [2,3,4]. The co-presence of Ca^++^ channel inhibitor SKF was not able to hinder the cytotoxic effects of HPF (Figure 5). In addition, TRPC6 expression level was not affected by HPF, both at early and late times of treatment (Figure 6). TRPC6 is a channel that allows Ca^++^ entry in cells. As reported by Shekhar et al. [67], the transcription factor CREB is a calcium sensor that can be activated by phosphorylation on its Ser133. Effectively, pCREB expression was increased by HPF in a concentration-dependent manner, whereas the expression of total CREB was unchanged or even decreased (Figure 6). These data are consistent with an HPF Ca^++^ dependence in our cell models, although these events do not hinder the antimelanoma effects of this natural compound. Recently, Scheuble et al. [68] reported that HPF stimulates the activity of the transcription factor AP1 via TRPC6. Indeed, in all melanoma cell lines, two components of AP1 complex, FRA1 and the phosphorylated and active form of cJun (pcJun), increased their expression at early times after HPF administration (Figure 6). Subsequently, the expression of both FRA1 and pcJun were strongly decreased (Figure 6), suggesting that the activation occurred but was transient. 

Cytosolic concentrations of calcium and zinc can be increased by TRPC6-elicited calcium influx or by releasing them from the mitochondria stores, another recognized property of HPF [5,6,7]. The co-treatment of HPF with BAPTA and TPEN, able to chelate intracellular Ca^++^ and Zn^++^, respectively, did not hinder the ability of HPF to affect cell viability (Figure 5). 

Thus, other signaling pathways have been investigated. The activation of kinases ERK1/2 and AKT is very important for allowing a high rate of cell proliferation, especially in BRAF-mutated melanoma cells. BRAF- and MEK-inhibitors are highly effective targeted drugs in melanoma therapy, but they can induce drug resistance both in vitro and in patients [41]. As expected, A375, FO-1 and SK-Mel-28 cell lines showed activated pro-survival kinases ERK1/2 and AKT in control cells. In contrast with data obtained in other tumor cells [30,56], HPF cannot decrease the activity of these kinases, at least not with 24 h of treatment (Figure 6). The exception is the FO-1 cell line, in which only pERK1/2 expression was decreased.

HPF, instead, can block the activity of two transcription factors involved in the pro-survival signaling pathways NF-κB and STAT3. 

We and other authors have previously reported that HPF and SJW extract had the ability to hinder NF-κB activation [33,34,56,69,70]. Many data demonstrate that NF-κB can be a specific target for counteracting melanoma progression [58]. Indeed, BMS-345541, a selective inhibitor of NF-κB, is known to induce an in vitro inhibition of cell proliferation and induction of apoptosis in three melanoma cell lines [71]. Constitutive activation of STAT3 plays a vital role in the development of melanoma [72]. Remarkably, in a melanoma mouse model, the silencing of STAT3 expression can reverse the malignant phenotype [73]. Tyr705 phosphorylation of STAT3 is an activating event since it allows STAT3 translocation into the nucleus. HPF can block STAT3 tyrosine phosphorylation in all the BRAF-mutated melanoma cell lines after 24 h treatment without affecting the total STAT3 protein level (Figure 6). In a cancer context, no data are available in the literature on STAT3 and HPF. Recently, Zhang et al. [74] reported that STAT3 inhibition is involved in the protective mechanism of HPF against psoriasis, and we previously demonstrated that the impediment of STAT3 transcriptional activity, elicited by SJW extract pre-treatment, can protect mice lungs injured by carrageenan [75]. Considering that many activation-signaling pathways converge on STAT3, such as EGFR, vascular endothelial growth factor, interleukin 6, focal adhesion kinase, proto-oncogene tyrosine kinase Src and others, we can state that STAT3 is an important target in melanoma therapy, and its blockage by HPF could be a crucial event among all the effects triggered by this natural compound. 

Since HPF has been reported to affect mitochondrial membrane potential by acting as a protonophore [9], we investigated its effect on the expression of mitochondrial enzymes of the electron transport chain and of some enzymes belonging to the glucose metabolic pathway. 

PGC1α is a transcription factor activating mitochondria biogenesis, but its expression is significantly reduced in the presence of HPF (Figure 7). The expression level of several key members of internal mitochondrial membrane functional complexes is similarly lowered, such as complex III (UQCRC1) and complex IV (COX4) components and the subunit β of ATP synthase (ATP5B) (Figure 7). All data are also in line with a possible dysfunction of the electron transport chain and the oxidative phosphorylation. Remarkably, the intrinsic apoptotic pathway is triggered by mitochondria membrane permeabilization. Moreover, in cells treated with HPF, studies that measured both apoptosis activity and mitochondrial membrane potential found a negative correlation among them [26,28,30,32,63]. The low expression levels of mitochondrial membrane functional proteins could be part of a general mechanism by which HPF leads to a drop in mitochondrial potential and functionality.

Despite the activation of AMPK, some cytosolic enzymes of glucose metabolism decreased their expression with the HPF treatment. LDHA, PGM2 and the phosphorylated form of pyruvate kinase M2, the level of which is high in tumor cells, are all less expressed (Figure 7). Indeed, the expression of HIF1α, a master activator of glucose metabolism, is strongly inhibited by HPF (Figure 7).

Finally, a wound healing assay and colony formation in soft agar demonstrated the ability of HPF to reduce cell mobility and colony growth in melanoma cell lines (Figure 8). This could suggest that HPF is able to counteract the metastatic potential of melanoma cells, as has been reported for other tumor cells [36,76,77]. 

## 4. Materials and Methods

### 4.1. Cell Cultures

A375 (CRL-1619) and FO1 (CRL-12177) melanoma cell lines (ATCC, Manassas, VA, USA) were cultured at 37 °C in a humidified atmosphere of 5% CO_2_, in the presence of high glucose Dulbecco’s modified Eagle Medium (DMEM, Gibco, BRL Invitrogen Corp., Carlsbad, CA, USA), supplemented with 10% heat-inactivated fetal bovine serum (FBS; Gibco, BRL Invitrogen Corp., Carlsbad, CA, USA) and with 1% antibiotic antimycotic solution (Gibco, BRL Invitrogen Corp., Carlsbad, CA, USA). 

SK-Mel-28 (HTB-72), MeWo (HTB-65) melanoma cell lines and normal human epidermal melanocytes (NHEM, PCS-200-013) (ATCC, Manassas, VA, USA) were grown in Roswell Park Memorial Institute 1640 medium (RPMI-1640, Gibco, BRL Invitrogen Corp., Carlsbad, CA, USA) under the previously described conditions.

### 4.2. Melanoma Cell Treatments

Hyperforin-DCHA (*AG*-*CN2*-*0008,* AdipoGen Life Sciences, Fuellinsdorf, Switzerland) was dissolved in 100% dimethyl sulfoxide at a concentration of 5 mM. Many aliquots of the stock solution were stored at −20 °C, protected from the light. Cell lines were treated with different HPF concentrations (range 0.5–10 μM). At the end of each treatment, different types of assays were performed. Images of cells after 24 or 48 h of treatments were captured at 20× magnification with an inverted microscope (Axio Vert A1, Zeiss, Oberkochen, Germany). The TRPC6 Ca^++^ channel blocker, SKF 96,365 (CAY-10009312, Cayman Chemical, Ann Arbor, MI, USA) was used, as well as the chelators of Ca^++^ and Zn^++^, respectively, BAPTA-AM (CDX-B0285, Biomol, Hamburg, Germany) and N,N,N,N,-tetrakis(2-pyridylmethyl) (TPEN) (Sigma-Merck, Milan, Italy). 

### 4.3. Cell Viability Assay

A375, FO1, SK-Mel-28 and NHEM cells were seeded in 96-well plates (A375: 3.0 × 10^3^ cells/well; FO1, SK-Mel-28, MeWo and NHEM: 6.0 × 10^3^ cells/well). After 24 h, cells were treated with different concentrations of HPF and incubated for 24, 48 and 72 h. At the end of each treatment, cells were fixed by adding 25 µL/well of 50% (w/v) trichloroacetic acid directly into the culture medium. Plates were incubated at 4 °C for 1 h, washed 4 times with ddH_2_O and dried at room temperature (RT). Staining was performed by adding 50 µL/well of 0.04% (w/v) sulforhodamine B (SRB) sodium salt solution (Sigma-Aldrich, Milan, Italy). After 1 h incubation at RT, plates were rinsed with 1% acetic acid and air-dried. SRB was solubilized in 10 mM Tris base solution, pH 10.5 and Abs 540 nm, measured in the plate reader TECAN NanoQuant Infinite M200 Pro (Tecan Group Ltd., Männedorf, Switzerland). Six replicates for each condition/data point were performed.

### 4.4. Br-deoxy-Uridine Cell Proliferation Assay

Cell proliferation was assessed with a colorimetric immunoassay based on the measurement of Br-deoxy-uridine (BrdU) incorporation during DNA synthesis. A375, FO1 and SK-Mel-28 cells were cultured in a 96-well plate as previously described, and after 24 h, incubated with or without HPF. After 48 h of treatment, the cells were incubated for 8 h with BrdU labelling solution (BrdU Cell Proliferation Kit, Roche, Merck, Milan, Italy), and then fixed with 200 μL/well of fix/denaturing solution for 30 min at RT. After washing 3 times, the peroxidase goat anti-mouse IgG conjugate was added, and the plate was incubated for 90 min at RT. Three washes were performed again, then 100 μL/well of TMB peroxidase substrate solution was added and plates were incubated at RT until color development was sufficient for photometric detection (5–30 min). Then, the absorbance of the samples was measured at Abs 370 nm (reference wavelength: approx. 492 nm) in a Tecan NanoQuant Infinite M200 Pro plate reader (Tecan Group Ltd., Männedorf, Switzerland). After many measurements at various time points (e.g., 5, 10 and 20 min), the reaction was stopped with 25 μL/well of 1 M H_2_SO_4_ and was measured in a plate reader at 450 nm (reference wavelength: 690 nm).

### 4.5. Total Protein Extracts

Cells were seeded in 6 cm Petri dishes (A375: 150 × 10^3^ cells/dish; FO1 and SK-Mel-28: 300 × 10^3^ cells/dish). After 24 h, cells were treated or not treated with increasing concentrations of HPF, alone or in presence of SKF, BAPTA and TPEN inhibitors. After 2 and 24 h of treatment, cells were scraped using warm 1× sample buffer (2% SDS, 10% glycerol, 50 mM Tris-HCl, 1.75% β-mercaptoethanol and bromophenol blue) and boiled at 99 °C for 10 min. Total protein extracts were kept at −80 °C until use.

### 4.6. Western Blot Analysis

Protein extracts were electrophoresed in a 10–12% polyacrylamide SDS-PAGE. Proteins were then transferred to a polyvinylidene difluoride membrane (PVDF, Merck-Millipore, Milan, Italy) and membranes were blocked at RT with TBST (10 mM Tris-HCl pH 7.5, 100 mM NaCl, 0.1% Tween 20) containing 5% milk for 1 h. Thereafter, they were incubated on a shaker overnight at 4 °C, with a 5% BSA solution containing the primary antibody against AMPK (#2532, 1:1000), Bcl-2 (#15071, 1:1000), GAPDH (#2118, 1:1000), LDHA (#3582, 1:2000), pACC (Ser79) (#11818, 1:1000), pAKT (Thr308) (#13038, 1:1000), pAMPK (Thr 172) (#2531, 1:1000), pERK (#9101, 1:1000), pP53 (#9286, 1:1000), pP65-NF-κB (Ser 536) (#3033, 1:1000), pRb (#8516, 1:2000), Rb (#9309, 1:2000), pSTAT3 (#9145, 1:2000) (Cell Signaling Technology, Danvers, MA, USA); AKT (GTX121937, 1:4000), ATP5B (GTX132925, 1:3000), CDK4 (GTX102993, 1:3000), COX4 (GTX114330, 1:3000), CREB (GTX112846, 1:3000), Cyclin A2 (GTX103042, 1:3000), Cyclin D1 (GTX108624, 1:10000), ERK1/2 (GTX134462, 1:10000), FRA1 (GTX134242, 1:4000), HIF1α (GTX127309, 1:3000), LC3B (GTX127375, 1:2000), P21/Waf1 (GTX629543, 1:3000), p4EBP1 (GTX133181, 1:6000), 4EBP1 (GTX116315, 1: 4000), P65-NF-κB (GTX102090, 1:3000), PARP1 (GTX628836, 1:3000), pcJun (GTX133873, 1:3000), pCREB (GTX130379, 1:3000), PGM2 (GTX119168, 1:3000), pPKM2 (GTX133886, 1:3000), STAT3 (GTX104616, 1:3000), TRPC6 (GTX113859, 1:3000), UQCRC1 (GTX630393, 1:1000) (Genetex, Alton Parkway, Irvine, CA, USA); GPX4 (67763-1, 1:3000) (Proteintech, Manchester, UK); P53 (sc-263, 1:3000) (Santa Cruz Biotechnology, Dallas, TX, USA); PGC1α (PA5-38022, 1:1000) (Invitrogen, Thermo Scientific, Waltham, MA, USA); and Bcl-xL (ab77571, 1:1000) (Abcam, Cambridge, UK). Then, membranes were washed 3 times with TBST buffer for 30 min and incubated for 1 h with a horseradish peroxidase-conjugated secondary antibody (anti-rabbit or anti-mouse, 1:6000-1:4000, Cell Signaling Technology, Danvers, MA, USA). Membranes were washed again 3 times for 30 min with TBST buffer. The expression of each protein was normalized with GAPDH protein level unless otherwise stated. Immuno-detection was carried out with an ECL kit (Merck-Millipore, Milan, Italy) and the chemiluminescence signals were visualized with ChemiDoc (Bio-Rad, Hercules, CA, USA).

### 4.7. Wound-Closure Cell Migration Assay

A375, FO1 and SK-Mel-28 cell migration was assessed by performing the wound-closure cell migration assay also known as a scratch test. Cells were seeded in a 12-well plate (A375: 150 × 10^3^ cells/well, FO1 and SK-Mel-28: 4 × 10^5^ cells/well). Once the cells had reached confluence, the wells were washed twice with PBS and once with complete medium. Then, the monolayers were scratched with a sterile pipette tip. To remove detached cells, wells were washed with a complete medium and refilled with fresh complete medium with or without HPF added. The cells were incubated for 24 h at 37 °C, in a humidified atmosphere with 5% CO_2_. Images of cell movement were captured every 4 h using an inverted microscope (Axio Vert A1, Zeiss, Oberkochen, Germany). The acquired images were further quantitatively analyzed by using ImageJ computing software, MRI Wound Healing Tool. 

### 4.8. Colony Formation Assay in Soft Agar

Anchorage-independent growth of A375, FO-1 and SK-Mel-28 melanoma cells was analyzed by colony formation in soft agar, as previously described [78]. Firstly, the bottom layer of 6 well-plates was filled with 1% low gelling temperature agarose (Sigma-Merck, Milan, Italy) dissolved in 2× DMEM, 20% FBS and 2% antibiotic, antimycotic solution. Thereafter, 0.6% low gelling temperature agarose dissolved in 2× DMEM, 20% FBS and 2% antibiotic, antimycotic solution, together with treated or not-treated cells (5000 cells/well) was placed over the 1% agarose layer. An amount of 100 μL–200 μL of fresh media were added to every well twice a week. After 15–21 days, the cell colonies that were formed were observed under an inverted microscope (Axio Vert A1, Zeiss, Oberkochen, Germany).

### 4.9. Statistics

All the results are reported as a mean value ± standard deviation (S.D.). Differences were analyzed with GraphPad Prism statistical program, using an unpaired, two-tailed Student’s *t*-test. A *p*-value less than 0.05 (*) or less than 0.01 (**) was considered to be statistically significant. For each type of experiment, a minimum of 3 independent biological replicates were performed. The normal distribution of data was tested using the Shapiro–Wilk test.

## 5. Conclusions

In conclusion, HPF was shown to be a potent natural compound able to hinder the expression and function of different key proteins involved in pro-survival and pro-metastatic signaling of BRAF-mutated melanoma cells.

This study, far from being comprehensive, aims to provide evidence of pleiotropic effects of this interesting natural compound in highly malignant melanoma cells, in order to identify possible molecular mechanisms that will be studied more thoroughly in the future.

Additionally, an overview of HPF’s mechanism of action in melanoma cells can pave the way for further studies to associate HPF with conventional antitumor drugs seeking synergistic effects.

## Data Availability

Not applicable.
